# Dacomitinib-Induced Paronychia Associated With PRIDE Syndrome in a Patient With Non-Small Cell Lung Cancer

**DOI:** 10.7759/cureus.85774

**Published:** 2025-06-11

**Authors:** Geethanjali Sahadevan, Divyashanthi CM, Kalaimani Sivamani

**Affiliations:** 1 Dermatology, Jawaharlal Institute of Postgraduate Medical Education & Research, Karaikal, IND; 2 Pharmacology, Jawaharlal Institute of Postgraduate Medical Education & Research, Puducherry, IND; 3 Internal Medicine, Jawaharlal Institute of Postgraduate Medical Education & Research, Puducherry, IND

**Keywords:** dacominitib, egfri, nsclc, paronychia, pride syndrome

## Abstract

Epidermal growth factor receptor (EGFR) inhibitors are essential for treating non-small cell lung cancer (NSCLC) with EGFR mutations, but they frequently cause cutaneous toxicities collectively referred to as papulopustules and/or paronychia, regulatory abnormalities of hair growth, itching, and dryness due to epidermal growth factor receptor inhibitors (PRIDE) syndrome. This case describes a 55-year-old male with advanced NSCLC who developed toe-predominant paronychia, a papulopustular rash localized to the trunk and limbs, and diarrhea four to eight weeks after initiating first-line therapy with dacomitinib. Notably, causality was evaluated using multiple validated tools - Naranjo, WHO-UMC, and Liverpool Causality Assessment Tool - all of which indicated a “probable” relationship. Importantly, these adverse effects were managed symptomatically without the need for discontinuation or dose reduction of dacomitinib. This case contributes to the literature by documenting an atypical anatomical distribution (toe-predominant), early onset, and successful continuation of therapy, highlighting the importance of early recognition, pharmacologic assessment, and adverse event reporting to optimize the safety of EGFR inhibitors in clinical practice.

## Introduction

Non-small cell lung cancer (NSCLC) accounts for approximately 85% of all lung cancer cases. In Asian populations, particularly in countries such as China and India, the prevalence of epidermal growth factor receptor (EGFR) mutations ranges from 30% to 50%, which is significantly higher than in Western populations [1]. This region-specific data underscores the importance of prioritizing EGFR-targeted therapies. Dacomitinib, a second-generation irreversible pan-human EGFR tyrosine kinase inhibitor, has been shown to improve progression-free survival in patients with EGFR-mutant NSCLC but is frequently associated with cutaneous adverse effects collectively known as papulopustules and/or paronychia, regulatory abnormalities of hair growth, itching, and dryness due to epidermal growth factor receptor inhibitors (PRIDE) syndrome [2,3]. Among these, paronychia occurs in 5-20% of patients and typically affects the fingers, especially the thumbs [4]. This report presents a clinically significant and atypical case of toe-predominant paronychia that developed within four weeks of initiating dacomitinib, earlier than typically reported. The causality of the reaction was supported by multiple validated tools (Naranjo, WHO-UMC, and Liverpool Causality Assessment Tool, LCAT), and the adverse effect did not require discontinuation of therapy. This case contributes to the literature by documenting an unusual anatomical distribution and early onset, highlighting the importance of early recognition, multidisciplinary management, and pharmacovigilance in optimizing patient outcomes.

## Case presentation

A 55-year-old male with stage IV NSCLC, a 20 pack-year smoking history, and well-managed comorbidities - hypertension (controlled with amlodipine) and type 2 diabetes mellitus (managed with glimepiride) - was initiated on dacomitinib 45 mg once daily. Four weeks after starting therapy, he reported painful toenail swelling, prompting a dermatology consultation at six weeks post-initiation.

**Figure 1:**
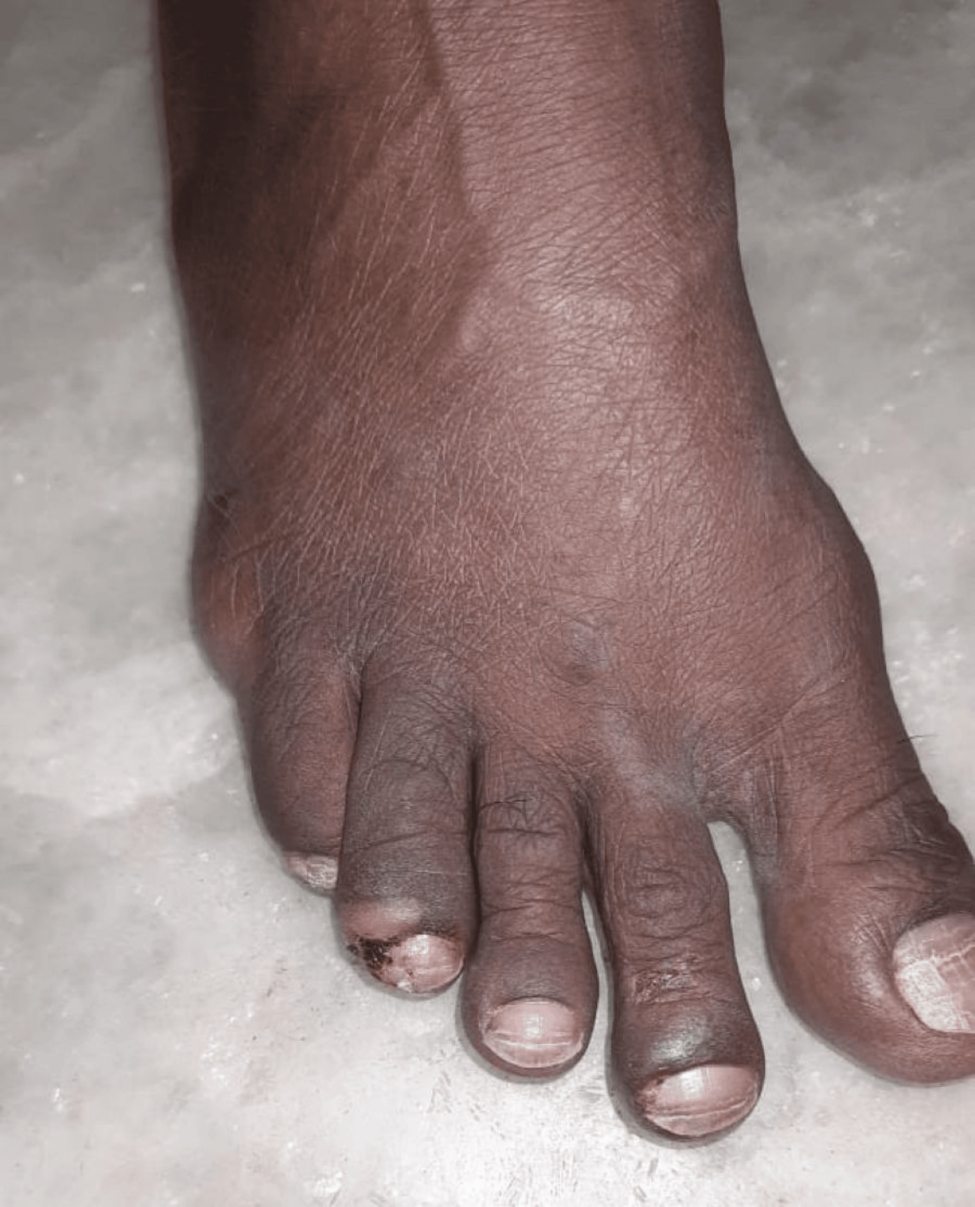
Paronychia of the right fourth toe

**Figure 2:**
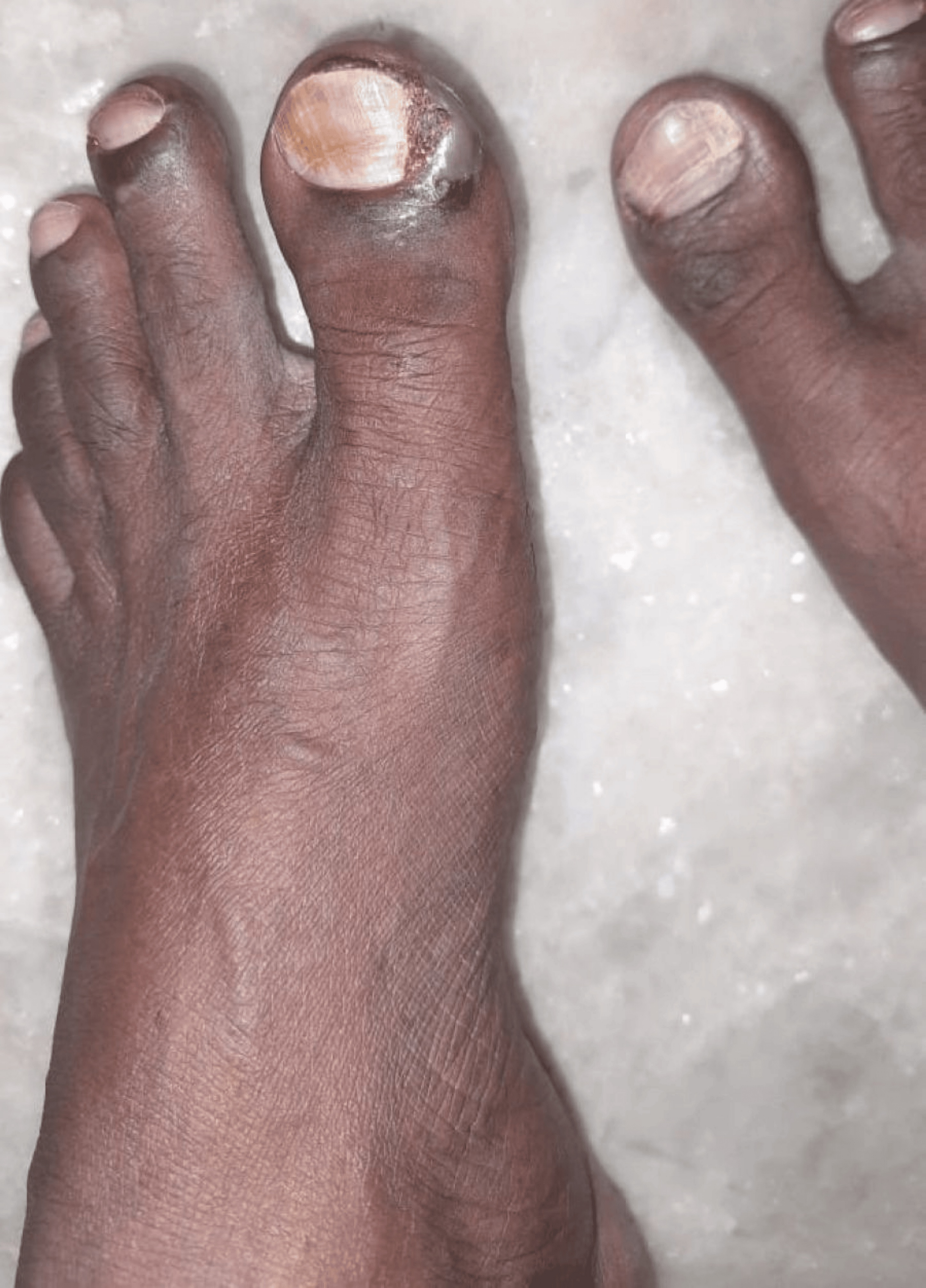
Paronychia of the left great toe with dry skin

Clinical examination revealed paronychia with onycholysis and periungual erythema, predominantly affecting the toes (Figure 1, Figure 2), along with a papulopustular rash on the trunk and lower limbs and mild diarrhea - features consistent with PRIDE syndrome. Notably, hair abnormalities and itching were absent. However, given that PRIDE syndrome encompasses a spectrum of cutaneous toxicities, the presence of three classical features - papulopustular rash, paronychia, and dryness - with a characteristic onset timeline supported the diagnosis. The clinical timeline included an NSCLC diagnosis six months prior, symptom onset at four weeks post-dacomitinib initiation, dermatology consultation within one week of symptom development, and documented improvement two weeks after intervention with continued monitoring.

**Figure 3:**
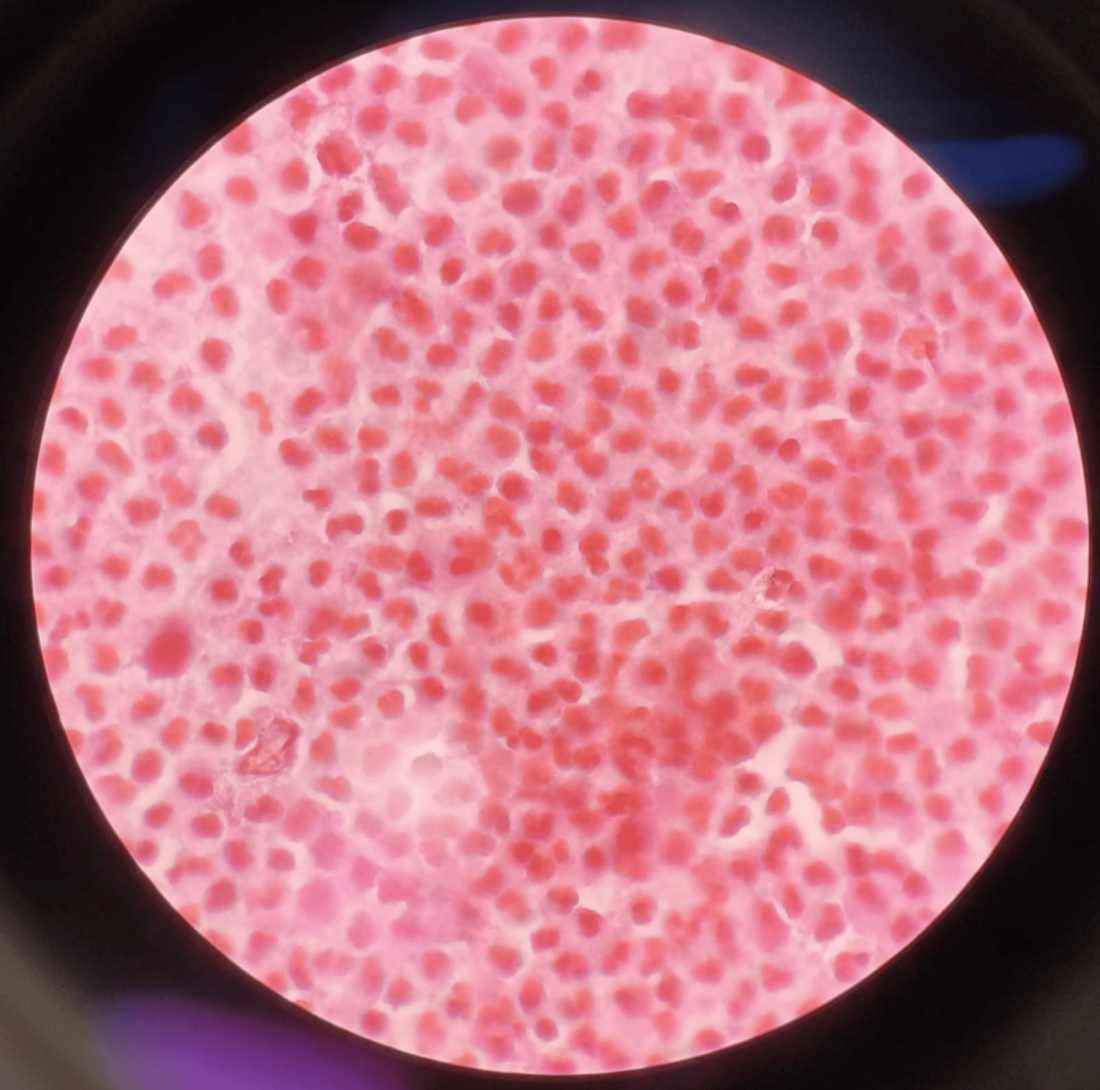
Gram stain of aspirate showing absence of bacterial organisms

PRIDE syndrome was diagnosed clinically based on the temporal association with dacomitinib and characteristic findings. There was no evidence of bacterial superinfection in the paronychia, as confirmed by a negative Gram stain and culture (Figure 3), and routine blood counts and inflammatory markers were within normal limits, ruling out alternative causes.

Causality was confirmed using multiple pharmacological assessment tools. The Naranjo Adverse Drug Reaction Probability Scale yielded a score of 7 (“probable”), based on symptom onset after drug initiation, improvement with treatment, the absence of alternative causes, and alignment with known toxicity profiles. The WHO-UMC Causality Assessment Scale also classified the reaction as “probable/likely,” supported by a clear temporal relationship, symptom resolution following intervention, and a plausible mechanism of action. The LCAT likewise indicated “probable” causality, consistent with dacomitinib’s established toxicity profile.

The Schumock and Thornton Preventability Scale classified the reaction as “not preventable,” reflecting the intrinsic nature of the adverse effect and the absence of modifiable risk factors or prescribing errors. Dacomitinib’s irreversible EGFR inhibition, which disrupts nail matrix cell migration, along with its prolonged half-life (~70 hours), were considered contributing factors.

Management focused on symptomatic relief without discontinuing dacomitinib. Paronychia was treated with paracetamol (500 mg as needed), doxycycline (100 mg twice daily for two weeks), antiseptic soaks, and emollients. The rash was managed with topical emollients, while diarrhea was controlled with loperamide as needed. Two weeks after initiating supportive treatment, the paronychia, rash, and diarrhea had improved, allowing continuation of dacomitinib at the full dose (45 mg daily), with maintained control of NSCLC.

## Discussion

Dacomitinib’s efficacy in EGFR-mutant NSCLC is well established; however, cutaneous toxicities, affecting approximately 80% of patients, pose significant clinical challenges [2]. In this case, the presentation of toe-predominant paronychia at four weeks is atypical, as EGFR inhibitor-related paronychia commonly involves the thumbs and tends to emerge after six to eight weeks of therapy [3]. Causality assessments strongly support a link between dacomitinib and the observed paronychia, consistent with its mechanism of EGFR inhibition, which disrupts nail matrix cell proliferation and induces sterile inflammation [5]. The association was confirmed using multiple standardized tools: the Naranjo scale (score: 7 - probable) [6], the WHO-UMC scale (probable/likely) [7], and the LCAT (probable) [8].

The Schumock and Thornton Preventability Scale classified the reaction as not preventable, indicating that the adverse event was intrinsic to the drug’s pharmacologic profile and not due to prescribing errors or modifiable factors, such as footwear-induced pressure [9]. However, emerging literature suggests that prophylactic interventions may reduce the severity of such toxicities, pointing to possible avenues for future clinical practice [10].

Compared with prior reports, this case's toe predominance contrasts with the predominantly finger (especially thumb) involvement noted in a 2022 meta-analysis, where paronychia was observed in 17.4% of patients receiving EGFR inhibitors [4]. A 2023 study evaluating dacomitinib’s long-term safety reported a 20% incidence of paronychia but did not specify anatomical distribution, highlighting the uniqueness of the current case [11]. The early onset at four weeks aligns with a 2021 review noting that second-generation tyrosine kinase inhibitors (TKIs) can cause cutaneous toxicities within one to two months, likely due to their irreversible binding and prolonged half-life (~70 hours) [12].

Toe involvement may be influenced by patient-specific factors, such as mechanical stress from footwear or regional microcirculatory differences. Genetic predispositions, such as EGFR polymorphisms, remain underexplored and represent an important area for future investigation [5].

Management in this case involved doxycycline, leveraging its anti-inflammatory properties, in accordance with current guidelines recommending tetracyclines for EGFR inhibitor-related skin toxicities [11]. The patient’s rapid clinical improvement within two weeks mirrors findings from a 2022 study in which doxycycline reduced paronychia severity in 70% of patients within three weeks [10]. Alternative interventions, such as topical beta-blockers (e.g., betaxolol), have shown promise in reducing paronychia-associated inflammation. A 2023 case series reported symptom resolution in 80% of patients using such therapies [13]; however, these remain investigational and are not part of standard treatment protocols, supporting the “not preventable” classification in this case.

The broader implications of this case extend to pharmacovigilance and clinical practice. Reporting the adverse drug reaction to the Pharmacovigilance Programme of India contributes to national and global databases, such as VigiBase, which are essential for post-marketing surveillance of newer agents like dacomitinib. A 2021 study emphasized that underreporting of dermatologic toxicities in Asian populations limits accurate safety profiling, highlighting the importance of such case reports [11].

This case also reinforces the value of patient education on early symptom recognition and the importance of multidisciplinary collaboration among oncologists, dermatologists, and pharmacologists. Such an approach is critical to managing toxicity, maintaining treatment adherence, and ultimately improving progression-free survival in NSCLC [2].

Limitations of this case include the absence of long-term oncologic outcomes, which could provide insights into the efficacy-toxicity balance of continued dacomitinib therapy. Additionally, the management approach was reactive rather than prophylactic. Nevertheless, emerging evidence suggests that preemptive strategies, such as low-dose doxycycline or routine emollient use, can reduce toxicity incidence by up to 30%, as reported in a 2022 trial [10], although these measures are not yet standardized.

Future research should focus on controlled trials evaluating prophylactic interventions and identifying predictive biomarkers, such as EGFR expression in skin tissues, that could anticipate individual toxicity risk. Genetic studies may also clarify why some patients, like the one described here, experience uncommon manifestations such as toe-predominant paronychia, possibly due to regional keratinocyte sensitivity or environmental exposures.

## Conclusions

This case highlights a rare presentation of toe-predominant paronychia as part of PRIDE syndrome induced by dacomitinib, supported by confirmatory causality assessments (Naranjo, WHO-UMC, and LCAT). Effective management without therapy discontinuation demonstrates the feasibility of continuing EGFR TKI treatment when adverse effects are promptly recognized and addressed. Reporting such cases enhances real-world pharmacovigilance and contributes to region-specific therapeutic guidance. Future research should prioritize prophylactic strategies and biomarker discovery to better anticipate and manage EGFR inhibitor-related toxicities.
